# A Cost-Effective Approach to Optimizing Microstructure and Magnetic Properties in Ce_17_Fe_78_B_6_ Alloys

**DOI:** 10.3390/ma10080869

**Published:** 2017-07-28

**Authors:** Xiaohua Tan, Heyun Li, Hui Xu, Ke Han, Weidan Li, Fang Zhang

**Affiliations:** 1 Institute of Materials, School of Materials Science and Engineering, Shanghai University, Shanghai 200072, China; liheyunde@126.com (H.L.); huixu8888@shu.edu.cn (H.X.); lwdhljj315@163.com (W.L.); 2 National High Magnetic Field Laboratory, Florida State University, 1800 E. Paul Dirac Drive, Tallahassee, FL 32310, USA; 3 Tescan China, Shanghai 201112, China; fang.zhang@tescanchina.com

**Keywords:** rare earth alloys, melt-spinning, quenching parameters, magnetic property, microstructure

## Abstract

Optimizing fabrication parameters for rapid solidification of Re-Fe-B (Re = Rare earth) alloys can lead to nanocrystalline products with hard magnetic properties without any heat-treatment. In this work, we enhanced the magnetic properties of Ce_17_Fe_78_B_6_ ribbons by engineering both the microstructure and volume fraction of the Ce_2_Fe_14_B phase through optimization of the chamber pressure and the wheel speed necessary for quenching the liquid. We explored the relationship between these two parameters (chamber pressure and wheel speed), and proposed an approach to identifying the experimental conditions most likely to yield homogenous microstructure and reproducible magnetic properties. Optimized experimental conditions resulted in a microstructure with homogeneously dispersed Ce_2_Fe_14_B and CeFe_2_ nanocrystals. The best magnetic properties were obtained at a chamber pressure of 0.05 MPa and a wheel speed of 15 m·s^−1^. Without the conventional heat-treatment that is usually required, key magnetic properties were maximized by optimization processing parameters in rapid solidification of magnetic materials in a cost-effective manner.

## 1. Introduction

Re_2_Fe_14_B-type permanent magnets (Re = rare earth metals) are essential components in many electric machines and have been widely-used in various fields due to their outstanding hard magnetic properties [1,2]. The rapid solidification technique of melt-spinning, with cooling rates up to 10^4^–10^6^ K·s^−1^, is one of the most important techniques for producing Re-Fe-B alloys with homogeneous nanostructure and desirable hard magnetic properties [3]. In this process, molten alloy is ejected onto a rotating metal chill (or wheel) of much larger thermal mass and rapidly quenched to produce thin ribbons, typically a few centimeters wide and a few microns thick. The experimental challenge is to better understand the processing conditions required to obtain a homogeneous microstructure. Researchers have found fabrication parameters, such as wheel speed and chamber gas pressure, have significant effects on the microstructure and, consequently, the magnetic properties of the quenched ribbons. For example, when the wheel speed is lower than an optimum wheel speed (i.e., under-quenched condition), inhomogeneous microstructure may form. Inhomogeneous microstructure refers to the scale of structure in a sample varying from 100 nm to 10 μm [4]. On the other hand, when the wheel speed is higher than the optimum wheel speed (over-quenched condition), larger fractions of amorphous phase are obtained [5]. The melt-spinning, however, is probably not suitable to make thin films below 1 μm for studying the nanocrystalline or amorphous phase. To make the materials with thickness below 1 μm, deposition methods are usually used [6].

The fabrication process related to melt-spinning for making Re-Fe-B can be subdivided into direct and two-step methods. In the direct method, the crystallized phases form directly from the melt without conventional heat-treatment. In the two-step method, an amorphous or over-quenched precursor is crystallized by subsequent heat-treatment. An optimum wheel speed in the direct method could directly lead to more uniform nanostructure and enhanced magnetic properties than that in the two-step method [7]. However, a major disadvantage of the direct method is its narrow process window. Out of this window, it has a tendency to produce inhomogeneous and undesirable microstructures, resulting in a wide variation in the magnetic properties [8]. Furthermore, in Nd-Fe-B melt-spun ribbons, it was reported that the reduction in the chamber pressure could effectively prevent the formation of gas pockets at the roll-contacted surfaces of ribbons, resulting in a uniform microstructure [9,10]. Therefore, optimization of the processing parameters that control the microstructure is critical to improving the magnetic properties of Re-Fe-B alloys.

In recent years, there has been great effort in searching for economically more attractive permanent magnet materials because of price volatility of key rare-earth elements, such as Nd and Dy [11,12,13]. In particular, researchers reported that Ce_17_Fe_78_B_6_ alloy prepared by the two-step method showed hard magnetic behavior at room temperature [14]. The properties of this alloy had reached remanence (*B_r_*) of 0.49 T, intrinsic coercivity (*H_c_^i^*) of 494 kA·m^−1^, and energy product ((*BH*)*_max_*) of 32.6 kJ·m^−3^. Therefore, Ce_2_Fe_14_B-typed alloys are suitable alternative permanent magnetic materials, as Ce is both abundant and low cost [15,16,17]. The intermediate heat treatment in the two-step method, however, adds to the cost. In this work, we investigated the effects of fabrication parameters (chamber pressure and wheel speed) on both microstructure and magnetic properties of Ce_17_Fe_78_B_6_ ribbons produced by a new direct method without heat-treatment. By adjusting only the casting parameters, homogeneous nanocrystalline microstructure and enhanced magnetic properties were obtained. Moreover, we present a relationship between the chamber pressure and the wheel speed, which were used to modify both the microstructure and the magnetic properties. Optimized chamber pressure and wheel speed led to homogenous microstructure with dispersed Ce_2_Fe_14_B and CeFe_2_ nanocrystals. At chamber pressure of 0.05 MPa and wheel speed of 15 m·s^−1^, our direct approach produced 16% higher (*BH*)*_max_* (38 kJ·m^−3^) than that produced by two-step method.

## 2. Materials and Methods

**Figure 1:**
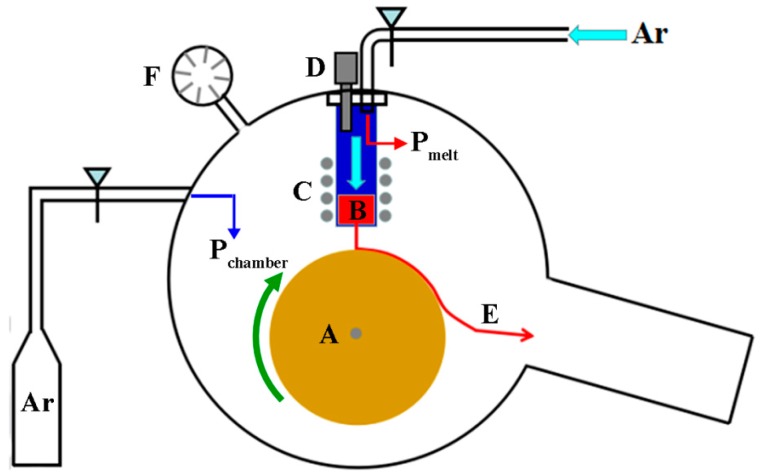
Schematic illustration of the melt-spinning process installed an infrared thermometer. (**A**): Cu wheel; (**B**): melt; (**C**): induction heater coil; (**D**): infrared thermometer; (**E**): ribbon; (**F**): vacuum meter.

Ingots with nominal composition Ce_17_Fe_78_B_6_ were prepared by arc-melting pure metals Ce, Fe, and Fe-B alloy in an argon atmosphere. Ingots were re-melted four times for homogenization. A melt-spinning process was employed (see Figure 1). The ejection pressure *P_eject_* is the difference between the inert gas applied to the melt in the quartz crucible, *P_melt_*, and the chamber pressure, *P_chamber_*. That is, *P_eject_* = *P_melt_* − *P_chamber_*. The *P_eject_* was maintained at 0.09 MPa by argon. A small portion of an ingot weighing about 5 g was re-melted in a quartz nozzle and ejected onto a rotating copper wheel through an orifice of diameter of 0.8 mm. During melt-spinning, the distance between the orifice and the copper wheel surface was maintained at 8 mm. An infrared thermometer located near the crucible was used to monitor the quenching temperature, which was maintained at 1588 K ± 5 K. The chamber pressure during melt-spinning was 0.02 MPa, 0.05 MPa, and 0.07 MPa. In Table 1, samples in group P were numbered depending on pressure in a range of 0.02–0.07 MPa, and samples in group W were numbered depending on wheel speed in a range of 8–25 m·s^−1^. The thickness of the ribbons was measured by a micrometer for five times and given as an average value (see Table 1). The magnetic properties of the ribbon samples were measured using a Lake Shore 7407 vibrating sample magnetometer (VSM) (Lake Shore Cryotronics, Westerville, OH, USA) with a maximum applied field of 1.8 T. At each experimental condition, six samples were measured and the results were consistent and reproducible at the chamber pressure in the range of 0.05–0.07 MPa, and the wheel speed in the range of 15–22 m·s^−1^. X-ray powder diffraction (XRD) patterns were recorded in a D/max-2550 diffractometer (Rigaku Corporation, Akishima-Shi, Tokyo, Japan) with Cu Kα radiation. The Rietveld refinement of diffraction data was carried out using Jana2006 software [18]. Cross-section samples from the ribbons were prepared using an IM4000 ion milling system (Hitachi, Tokyo, Japan), and were examined by a GAIA3 scanning electron microscope (SEM) (Tescan, Brno, Czech Republic). Transmission electron microscopy (TEM) was performed using a JEM 2010F (JEOL Ltd., Akishima, Tokyo, Japan), with a field emission electron gun operating at 200 kV. Plan-view TEM samples close to the wheel surface were prepared by grinding ribbons to a thickness of 20 μm and subsequent electropolishing in a solution of 5% HClO_4_ + 95% ethanol at 20 V at 243 K ± 5 K. 

## 3. Results

Magnetic properties were sensitive to both chamber pressure and wheel speed. When pressure was increased from 0.02 MPa to 0.05 MPa, energy product ((*BH*)*_max_*) was increased from 22 kJ·m^−3^ to 38 kJ·m^−3^, up by 73% (see samples in group P in Table 1). Further increasing the chamber pressure to 0.07 MPa marginally increased intrinsic coercivity (*H_c_^i^*), but decreased remanence (*B_r_*) and (*BH*)*_max_*. When wheel speed was increased, *H_c_^i^* was initially increased, but once *H_c_^i^* reached the maximum value of 496 kA·m^−1^, it began to decrease significantly despite further increases in wheel speed (see samples in group W in Table 1). The optimized magnetic properties, i.e., *H_c_^i^* = 491 kA·m^−1^, *B_r_* = 0.49 T, and *(BH)_max_* = 38 kJ·m^−3^ were achieved at a chamber pressure of 0.05 MPa and a wheel speed of 15 m·s^−1^.

**Figure 2:**
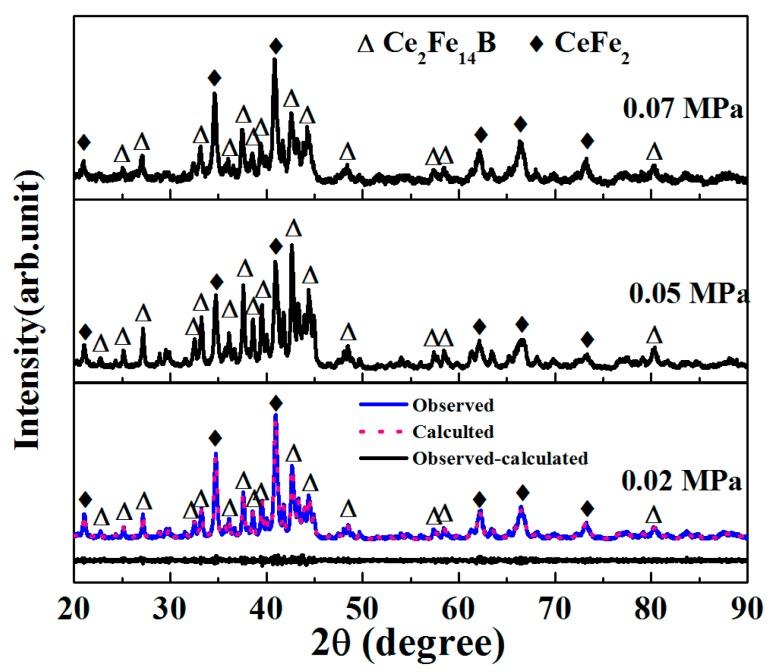
X-ray diffraction patterns of Ce_17_Fe_78_B_6_ ribbons prepared at a constant wheel speed of 15 m·s^−1^, but at chamber pressures of 0.02, 0.05 and 0.07 MPa.

XRD patterns of Ce_17_Fe_78_B_6_ powder samples are shown in Figure 2. For sample P2-15, Ce_2_Fe_14_B, and CeFe_2_ phases were observed. In samples made by higher chamber pressure, same crystalline phases were present. However, the relative intensities of peaks from Ce_2_Fe_14_B and CeFe_2_ phases changed with chamber pressure. That is, the relative peak intensities of Ce_2_Fe_14_B phase increased at 0.05 MPa, whereas decreased at 0.07 MPa. The relative peak intensities of CeFe_2_ phase showed the opposite trend. This result indicates that the volume fractions of the crystalline phases change with chamber pressure. Mass fractions of Ce_2_Fe_14_B and CeFe_2_ were obtained by an analysis of diffraction data using Jana2006 software [18]. Volume fractions of both phases were estimated using corresponding density data (*ρ*(Ce_2_Fe_14_B) = 7.7 g·cm^−3^ and *ρ*(CeFe_2_) = 8.6 g·cm^−3^, see Table 1). The Curie temperature (*T_C_*) of CeFe_2_ is 230 K (below the room temperature) [19] and the *T_C_* of Ce_2_Fe_14_B is 425 K (above the room temperature) [20]. Consequently, we concluded that the Ce_2_Fe_14_B phase was the one responsible for hard magnetic behavior at room temperature. Even a small change of volume fraction of Ce_2_Fe_14_B phase can have a significant impact. The volume fraction difference of the Ce_2_Fe_14_B phase in samples P2-15 and P7-15, for example, was only 4%, while the value of (*BH*)*_max_* was increased by 41%. When the volume fraction of the Ce_2_Fe_14_B phase was increased by 14% (by comparing samples P2-15 and P5-15 in Table 1), *B_r_* and (*BH*)*_max_* increased by 23% and 73%, respectively. For wheel speed impact, a similar trend was observed. For example, the volume fraction difference of the Ce_2_Fe_14_B phase in sample W5-8 and W5-22 was only 8%, while the *H_c_^i^* of W5-22 was 2.6 times greater than that of W5-8 sample. This data suggests that the magnetic properties of as-spun Ce_17_Fe_78_B_6_ ribbons are related, not only to the volume fraction of the hard magnetic phase, but also to other features, such as microstructure. 

**Figure 3:**
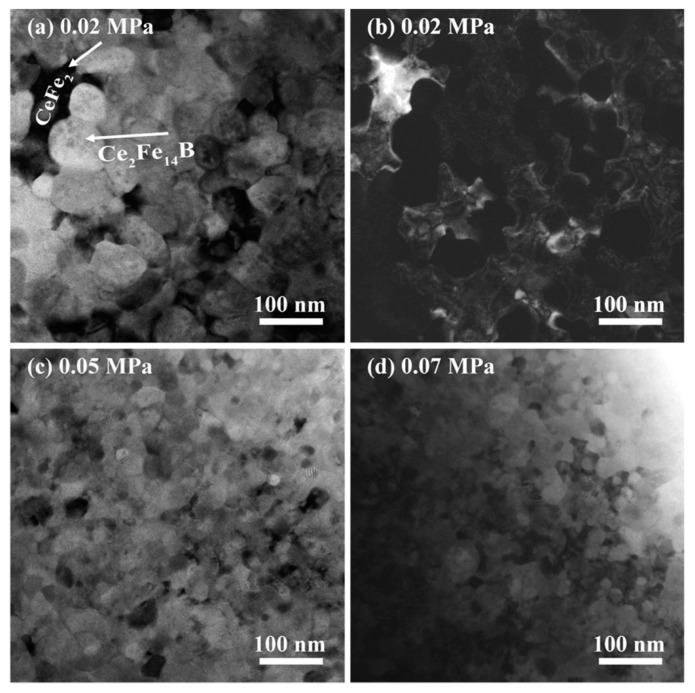
Transmission electron microscopy (TEM) images of Ce_17_Fe_78_B_6_ ribbons prepared at various chamber pressures at the wheel speed of 15 m·s^−1^: (**a**) bright field (BF) and (**b**) dark field (DF) images of samples made with pressure of 0.02 MPa; (**c**) 0.05 MPa; (**d**) 0.07 MPa. The arrows mark the formed phases. DF image in (**b**) shows CeFe_2_ phase.

TEM was used to probe the microstructure of as-spun Ce_17_Fe_78_B_6_ ribbons in group P. In sample P2-15 produced under low chamber pressure, both the CeFe_2_ phase and the Ce_2_Fe_14_B phase were observed (see Figure 3a). The grain size distribution was determined from the TEM images (Supplementary Figure S1 and Table S1). The average grain size of the Ce_2_Fe_14_B phase was 66 nm. The TEM dark-field image showed that irregularly shaped CeFe_2_ grains, averaging around 100 nm in size, were scattered among equiaxial Ce_2_Fe_14_B grains (see Figure 3b). In samples P5-15 and P7-15 produced under higher pressure, the average grain size of both CeFe_2_ and Ce_2_Fe_14_B decreased significantly to about 25 nm and 30 nm, respectively, though the shape of the grains changed only marginally (see Figure 3c,d). These results indicate that increasing chamber pressure leads to a refined microstructure in Ce_17_Fe_78_B_6_ ribbons, resulting in an improvement in magnetic properties. 

**Figure 4:**
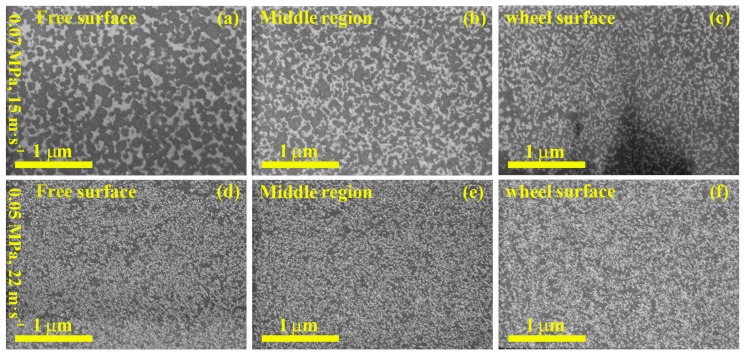
Cross-sectional back-scattered scanning electron microscope (SEM) images of as-spun Ce_17_Fe_78_B_6_ ribbons produced at the wheel speed of 15 m·s^−1^ and the chamber pressure of 0.07 MPa (**a**–**c**), and the ribbon spun at the wheel speed of 22 m·s^−1^ and the chamber pressure of 0.05 MPa (**d**–**f**). Both samples showed two different grey levels suggesting the presence of two phases, which was consistent with the XRD results. Because CeFe_2_ contains more Ce than Ce_2_Fe_14_B, the lighter contrasted regions were considered as CeFe_2_.

Samples P7-15 and W5-22, which exhibited exactly the same volume fraction of Ce_2_Fe_14_B and CeFe_2_, had similar magnetic properties (see Table 1), suggesting similar microstructures. This was confirmed by cross-sectional back-scattered SEM images (Figure 4). In sample P7-15, the grain size of both phases decreased from the free surface to the wheel surface (that is, the surface in contact with the copper wheel) (Figure 4a–c). Both phases were distributed uniformly. For sample W5-22, two phases with smaller grain size had a uniform distribution from the free surface to the wheel surface (Figure 4d–f). These TEM results indicate that chamber pressure and wheel speed may not be completely independent. Several different combinations may result in similar microstructure and magnetic properties.

## 4. Discussion

Although it is well-known that magnetic properties can be tuned by grain sizes and their distribution, researchers have been pursuing a cost-effective approach to refine and homogenize grain sizes. Researchers have reported, for example, that a reduction in chamber pressure can effectively prevent the formation of gas pockets at the roll-contacted surfaces of NdFeB-based melt-spun ribbons, resulting in a uniform microstructure [9,10]. However, our work demonstrated that in Ce_17_Fe_78_B_6_ ribbons, increasing, rather than decreasing, chamber pressure led to a more uniform microstructure, resulting in improved magnetic properties. Most previous researchers investigated only one parameter at a time, but we found that better optimization can be achieved by systematically adjusting two or more parameters at the same time. Samples P7-15 and W5-22, for example, exhibited close values of magnetic properties due to their similar microstructure. This appears to indicate a mutual dependency between chamber pressure and wheel speed.

In Kramer’s work [10], the relationship between ribbon thickness, *t*, and wheel speed, *v*, is given by

(1) $$t=c\sqrt{\left(a/\upsilon +b\right)\eta /\rho \upsilon }$$

where *c* is a constant, *b* is the melt stream diameter, *η* is the viscosity, *ρ* is the density, and *a* is a parameter which is a function of crucible pressure. 

Equation (1) can be rewritten as

(2) $$t^{2}=A\left(a/\upsilon^{2}+b/\upsilon \right)$$

where *A* = *c*^2^*η/ρ*, and *ρ* is a constant.

In our experiment, *b* was fixed and therefore a constant. The quenching temperature was maintained at 1588 K ± 5 K, assuming to be a constant *η*. Thus, parameter *A* is a constant. For the case of a constant chamber pressure, *a* is constant. The above analyses of Equation (2) led to the conclusion that ribbon thickness decreases with increasing wheel speed, as demonstrated by our experimental results (see Table 1). When wheel speed is fixed, however, chamber pressure impact can be reflected by variable *a*. The result for samples in group P shows that the ribbon thickness decreases with increasing chamber pressure (see Table 1). If Equation (2) is valid in a situation when pressure is a variable, parameter *a* needs to decrease with increasing chamber pressure. Here, we assume the parameter *a* is inversely proportional to chamber pressure, *P_chamber_*. Hence, the relationship between ribbon thickness, chamber pressure, and wheel speed can be expressed as

(3) $$t=\sqrt{A\left(k/P_{chamber}\upsilon^{2}+b/\upsilon \right)}$$

where *A*, *k* and *b*, are constants, and *k* = *a* × *P_chamber_*.

Equation (3) indicates that different combinations of chamber pressure and wheel speed might lead to a similar ribbon thickness.

Samples P7-15 and W5-22, were made by different combinations of experimental parameters, but their thickness sizes similar (27 μm and 26 μm, respectively). These two samples also show similar microstructure and, consequently, similar magnetic properties. Our results indicate that sample thickness is a critical *geometrical* parameter in determining both cooling rate and undercooling, which in turn govern microstructure and magnetic properties. The adjustment of above properties can be achieved by tuning both chamber pressure and wheel speed. These findings provide a guide for the preparation of alloys with desired magnetic properties and microstructure through adjusting fabrication parameters.

Herbst produced annealed Ce_17_Fe_78_B_6_ ribbons that had 88 vol % Ce_2_Fe_14_B phase [14]. In sample P5-15, the Ce_2_Fe_14_B volume fraction was 80%, lower than that in Herbst’s annealed sample. The (*BH*)*_max_* of 38 kJ·m^−3^ in P5-15, however, is about 16% higher than that in Herbst’s annealed sample (32.6 kJ·m^−3^). These results indicate that the magnetic properties of as-spun Ce_17_Fe_78_B_6_ ribbons are related not only to the volume fraction of hard magnetic Ce_2_Fe_14_B phase, but also to the microstructure. Refined and uniform microstructure can be obtained by adjusting chamber pressure and wheel speed without applying any heat treatment at all. Without heat treatment, our direct method uses less energy and resources, and is more cost-effective.

## 5. Conclusions

Using Ce_17_Fe_78_B_6_ alloys that had been fabricated by quenching without subsequent heat-treatment, we investigated the effects of chamber pressure and wheel speed on microstructure and magnetic properties. The results demonstrated that all directly-quenched samples exhibited hard magnetic behavior at room temperature. Good reproducibility of magnetic properties in as-spun ribbons was obtained at chamber pressure in a range of 0.05–0.07 MPa and wheel speed in a range of 15–22 m·s^−1^. The magnetic properties of as-spun Ce_17_Fe_78_B_6_ ribbons were governed by both microstructure and volume fraction of the Ce_2_Fe_14_B phase. Optimum magnetic properties, i.e., *H_c_^i^* = 491 kA·m^−1^, *B_r_* = 0.49 T and (*BH*)*_max_* = 38 kJ·m^−3^, and homogenous microstructure were both obtained at a chamber pressure of 0.05 MPa and a wheel speed of 15 m·s^−1^. A relationship was established between chamber pressure and wheel speed in rapid solidification. This work shed light on how to produce hard magnetic alloys by using melt-spinning alone, rather than along with more costly heat-treatment.

## Figures and Tables

**Table 1:** The intrinsic coercivity (*H_c_^i^*), the remanence (*B_r_*), energy product ((*BH*)*_max_*), the ribbon thickness (*t*), the volume fraction of Ce_2_Fe_14_B and CeFe_2_ phase of Ce_17_Fe_78_B_6_ alloy at various chamber pressures (*P_chamber_*) and wheel speeds (*v*).

Samples	*P_chamber_* (MPa)	*v* (m·s^−1^)	*t* (μm)	*H_c_^i^* (kA·m^−1^)	*B_r_* (T)	(*BH*)*_max_* (kJ·m^−3^)	Ce_2_Fe_14_B/CeFe_2_ Volume Fraction (%)
P2-15	0.02	15	42 ± 2	(0.40 ± 0.01) × 10^3^	0.40 ± 0.02	22 ± 2	69/31
P5-15	0.05	15	29 ± 2	491 ± 4	0.49 ± 0.01	38 ± 1	80/20
P7-15	0.07	15	27 ± 2	495 ± 6	0.43 ± 0.01	31 ± 2	72/28
W5-8	0.05	8	45 ± 2	(0.19 ± 0.02) × 10^3^	0.33 ± 0.02	7 ± 1	78/22
W5-22	0.05	22	26 ± 2	496 ± 6	0.42 ± 0.01	28 ± 1	72/28
W5-25	0.05	25	21 ± 2	74 ± 8	0.02 ± 0.01	<1	-

## Supplementary Materials

The following are available online at www.mdpi.com/1996-1944/10/8/869/s1. Figure S1: Grain size distribution of Ce_2_Fe_14_B and CeFe_2_ phases in as-spun Ce_17_Fe_78_B_6_ ribbons: (a,b) P2-15 sample; (c,d) P5-15 sample; and (e,f) P7-15 sample. Table S1: The grain size (*d*) of Ce_2_Fe_14_B and CeFe_2_ phases of Ce_17_Fe_78_B_6_ ribbons prepared at various chamber pressure (*P_chamber_*) and the wheel speed (*ν*) of 15 m⋅s^-1^.
