# Supplementary material for: A Cost-Effective Approach to Optimizing Microstructure and Magnetic Properties in Ce17Fe78B6 Alloys
Source: Materials (Basel). 2017 Jul 28;10(8):869. doi: 10.3390/ma10080869 (PMC5578235; doi:10.3390/ma10080869)
Supplement: Supplementary file 1 [file materials-10-00869-s001.pdf]

Supplementary Materials: A cost-effective approach to optimizing microstructure and magnetic properties in $Ce_{17}Fe_{78}B_6$ alloys

Xiaohua Tan, Heyun Li, Hui Xu, Ke Han, Weidan Li, Fang Zhang

Figure S1 shows the grain size distribution of $Ce_2Fe_{14}B$ and $CeFe_2$ phases in samples P2-15, P5-15 and P7-15. The average grain size of both phases decreased with an increase of chamber pressure.

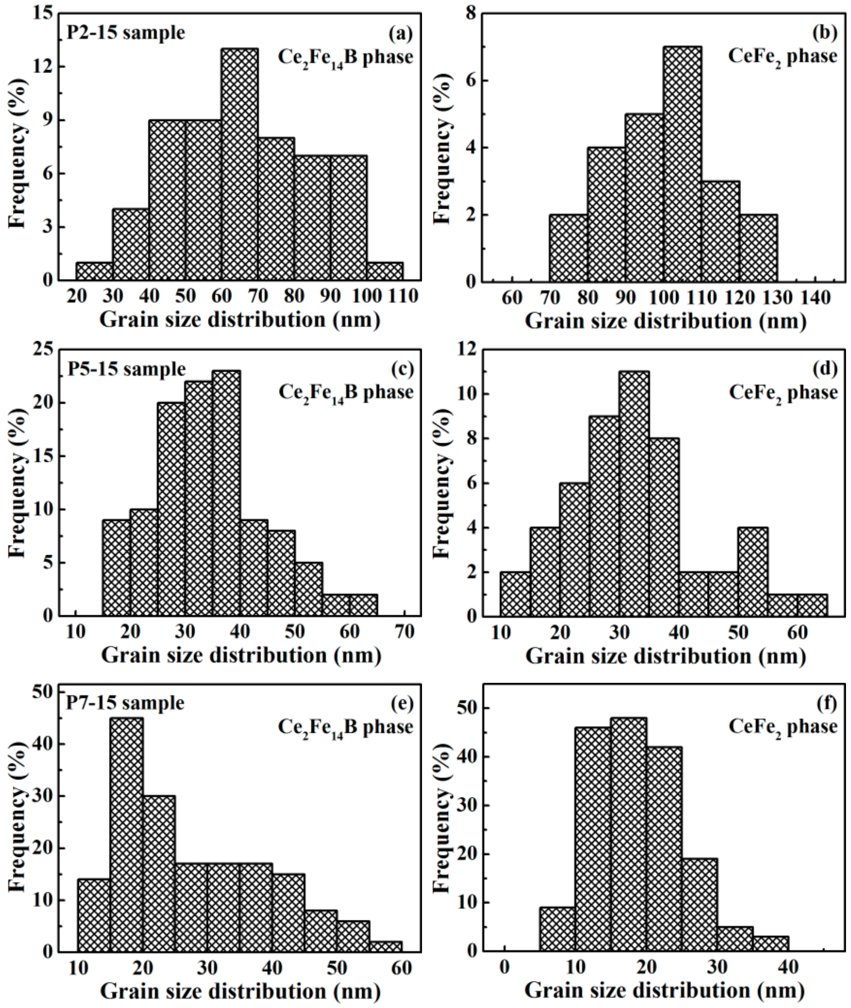

Figure S1. Grain size distribution of $Ce_2Fe_{14}B$ and $CeFe_2$ phases in as-spun $Ce_{17}Fe_{78}B_6$ ribbons: (a) (b) P2-15 sample, (c) (d) P5-15 sample, and (e) (f) P7-15 sample.

Table S1 The grain size (*d*) of $Ce_2Fe_{14}B$ and $CeFe_2$ phases of $Ce_{17}Fe_{78}B_6$ ribbons prepared at various chamber pressure ($P_{chamber}$) and the wheel speed (ν) of 15 m·s^{-1}.

Samples	$P_{chamber}$ (MPa)	ν (m·s^{-1})	$d_{Ce2Fe14B}$ (nm)	d_{CeFe2} (nm)
P2-15	0.02	15	66 ± 18	100 ± 13
P5-15	0.05	15	34 ± 9	32 ± 10
P7-15	0.07	15	27 ± 11	19 ± 6
